# Apparent diffusion coefficients of 31P metabolites in the human calf muscle at 7 T

**DOI:** 10.1007/s10334-023-01065-3

**Published:** 2023-02-08

**Authors:** Zhiwei Huang, Giulio Gambarota, Ying Xiao, Daniel Wenz, Lijing Xin

**Affiliations:** 1 Animal Imaging and Technology Core (AIT), Center for Biomedical Imaging (CIBM), Ecole Polytechnique Fédérale de Lausanne, Lausanne, Switzerland; 2 CIBM Center for Biomedical Imaging, EPFL CIBM-AIT, Station 6, CH-1015 Lausanne, Switzerland; 3 Faculty of Pharmacy, University of Rennes, Rennes, France

**Keywords:** Apparent diffusion coefficient, Phosphorous metabolites, ATP, PCr, DW-MRS

## Abstract

**Purpose:**

In this study, we aimed to measure the apparent diffusion coefficients (ADCs) of major phosphorous metabolites in the human calf muscle at 7 T with a diffusion-weighted (DW)-STEAM sequence.

**Methods:**

A DW-STEAM sequence with bipolar gradients was implemented at 7 T, and DW MR spectra were acquired in three orthogonal directions in the human calf muscle of six healthy volunteers (TE/TM/TR = 15 ms/750 ms/5 s) at three *b*-values (0, 800, and 1200 s/mm^2^). Frequency and phase alignments were applied prior to spectral averaging. Averaged DW MR spectra were analyzed with LCModel, and ADCs of ^31^P metabolites were estimated.

**Results:**

Four metabolites **(**phosphocreatine (PCr), adenosine triphosphate (ATP), inorganic phosphate (Pi) and glycerol phosphorylcholine (GPC)**)** were quantified at all *b*-values with mean CRLBs below 10%. The ADC values of PCr, ATP, Pi, and GPC were (0.24 ± 0.02, 0.15 ± 0.04, 0.43 ± 0.14, 0.40 ± 0.09) × 10^–3^ mm^2^/s, respectively.

**Conclusion:**

The ADCs of four ^31^P metabolites were successfully measured in the human calf muscle at 7 T, among which those of ATP, Pi and GPC were reported for the first time in humans. This study paves the way to investigate ^31^P metabolite diffusion properties in health and disease on the clinical MR scanner.

**Supplementary Information:**

The online version contains supplementary material available at 10.1007/s10334-023-01065-3.

## Introduction

^31^P MRS is a non-invasive technique for studying energy metabolism in vivo to track high-energy metabolites and to evaluate energy production and transport. Diffusion MRS allows the measurement of metabolite diffusion properties and thus provides insights into cellular compartmentation. Phosphocreatine (PCr), adenosine triphosphate (ATP), and inorganic phosphate (Pi) are the three main ^31^P MRS detectable metabolites which are essential for energy production and transport [1]. Another ^31^P metabolite glycerophosphocholine (GPC) is involved in cell membrane phospholipid metabolism and reflects malignant transformation of cells [2, 3]. Combining ^31^P and diffusion MRS to assess their diffusion properties could provide information regarding the diffusion-related energy supply mechanism and membrane kinetics in vivo.

The low concentration of phosphorous metabolites, low sensitivity of ^31^P nuclei, and high-gradient amplitude required (due to the low gyromagnetic ratio) are the main challenges for ^31^P diffusion MRS in humans. PCr has often been the main target for ^31^P diffusion MRS studies due to its dominant signal intensity [2–8]. To the best of our knowledge, most of these studies remained on the animal experiment phase. The diffusion coefficient of PCr has been measured in resting bullfrog biceps [10], goldfish white skeletal muscles [6, 8], and rat skeletal muscles [5, 7, 9]. Gabr et al. [4] reported for the first time diffusion properties of PCr in the human calf muscle at 3 T. In that study, a depth-resolved surface-coil spectroscopy (DRESS) sequence was implemented for a 1D localized measurement. Due to the low signal-to-noise (SNR), no phase correction was applied, which could render the measured values less reliable. Furthermore, due to the low concentration, short *T*_2_ relaxation time and J-coupling effects of ATP, its diffusion property has rarely been investigated. The diffusion coefficient of ATP has been previously measured in the skeletal muscle of rat [5] and bullfrog [10], however, it has never been measured in humans.

In this study, we aim to investigate the diffusion coefficients of major phosphorous metabolites (PCr, ATP, Pi and GPC) in the human calf muscle in vivo at 7 T. A diffusion-weighted (DW)-STEAM sequence with bipolar gradients was implemented to achieve 3D localized measurements. Diffusion gradients with opposite polarities were applied to eliminate the effect of cross-terms. To enable the measurement of apparent diffusion coefficients (ADCs) of ATP, a protocol with short echo time was used to minimize signal loss due to T_2_ relaxation and J-evolution. With improved sensitivity at 7 T [11], sufficient spectral SNR allows phase and frequency correction of each acquisition prior to spectra averaging to compensate for the effect of motion and improve the reliability of diffusion coefficient measurements.

## Methods

### MR sequence

**Fig. 1:**
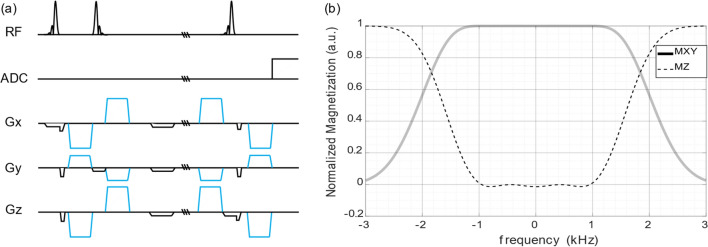
**a** The DW-STEAM pulse sequence. The diffusion gradients are depicted with blue lines; the localization and spoiler gradients are in black. **b** The excitation profile of the SLR and time-inverse SLR pulses (the second 90° RF pulse in (**a**)) simulated with Bloch equations

A DW-STEAM pulse sequence was implemented (Fig. 1a). To reduce echo time while keeping a large enough excitation spectral bandwidth, a 1600 μs long asymmetric Shinnar-Le Roux RF pulse (SLR pulse) and an inverse SLR pulse were implemented [12, 13]. The full-width half maximum bandwidth of the pulse is 3.26 kHz (Fig. 1b) (i.e., 27 ppm). Diffusion gradients were applied in three orthogonal directions [−0.5, 1, 1], [1, −0.5, 1], and [1, 1, −0.5]. To attenuate eddy currents induced by the diffusion gradients, bipolar gradients were implemented. Due to the existence of spoilers and localization gradients, cross-term interactions between them and diffusion gradients could arise, and thus bias the calculation of *b*-values, thus leading to the overestimation of diffusion coefficients. To eliminate the effect of cross-terms, diffusion gradients of opposite polarities were applied in each direction. Taken together, experiments along six directions were applied ([−0.5, 1, 1], [1, −0.5, 1], [1, 1, −0.5], [0.5, −1, −1], [−1, 0.5, −1], [−1, −1, 0.5]).

### In vitro MR experiments

To validate the DW-STEAM sequence, the ADCs of water, Pi, PCr, and ATP were measured in vitro at room temperature (~ 18℃). ^1^H experiments were carried out with a spherical phantom containing water (1L, pH = 7.0) using a single-channel quadrature transmit and a 32-channel receive coil (Nova Medical Inc., MA, USA), with TE/TM/TR = 15 ms/750 ms/40 s, number of averages (NA) = 4, and voxel size = 16 × 16 × 16 mm^3^. ^31^P experiments were carried out with a phantom containing 31 mM PCr, 31 mM ATP, 8 mM NaH_2_PO_4_ and 106 mM NaCl (0.5 L, pH = 7.2) using a home-built surface coil with a single ^31^P loop (diameter of 7 cm) and two quadrature ^1^H loops (diameter of 10 cm), with TE/TM/TR = 15 ms/750 ms/60 s, NA = 4, and voxel size = 40 × 46 × 46 mm^3^. For both experiments, the same sequence was used, with the carrier frequency set on water and PCr peak, respectively. The flip angle calibration of the RF pulses was performed with the same sequence without diffusion gradients by varying the transmitter voltage and selecting the one giving the highest signal intensity. Diffusion-weighted spectra at seven different *b*-values (0, 200, 400, 600, 800, 1000, 1200 s/mm^2^) were measured. All *b*-values *b*_diff_ were calculated without taking into accounts the spoilers and localization gradients. Thus, the *b*-values reported here were smaller than the actual *b*-values. Since the *b*-value originating by the spoilers and localization gradients was canceled when dividing the signal intensity by that of *b* = 0 s/mm^2^, the estimation of ADC values would not be biased, as shown in the following equation, where $\widetilde{{S}_{b}}$ and $\widetilde{{S}_{0}}$ are the signal intensity at calculated *b*-values *b* and 0 s/mm^2^, *S*_*0*_ is the signal intensity at actual *b*-value 0 s/mm^2^, *b*_other_ and *b*_diff_ are the *b*-values originating by the spoilers and localization gradients, and diffusion gradients, respectively, and *D* is the diffusion coefficient:

$$\ln \left( {\frac{{\widetilde{{S_{b} }}}}{{\widetilde{{S_{0} }}}}} \right) = \ln \left( {\frac{{S_{0} e^{{ - \left( {b_{{{\text{other}}}} + b_{{{\text{diff}}}} } \right)D}} }}{{S_{0} e^{{ - b_{{{\text{other}}}} D}} }}} \right) = - b_{{{\text{diff}}}} D$$

The maximum gradient amplitude (*b* = 1200 s/mm^2^) used was 49.1 mT/m, and the diffusion time was 757.5 ms.

### In vivo MR experiments

Six healthy subjects (18–40 years old, 3 males and 3 females) were scanned for in vivo ADC measurements. Informed consent forms were obtained from all subjects before the scan. The carrier frequency was set on the PCr peak. The MR spectra were acquired from a VOI of 60 mm × 100 mm × 120 mm containing the calf muscle. Voltage calibration of the RF pulses was done with the same sequence prior to the diffusion experiments to reach the highest signal intensity. A TE of 15 ms was used in this study as a compromise between the maximum achievable *b*-values and the short echo time required for sufficient detection of ATP, the resonances of which decay rapidly due to *T*_2_ relaxation time and J-evolution. Localized diffusion-weighted MR spectra at three *b*-values (0, 800, 1200 s/mm^2^) were acquired per diffusion direction (TE/TM/TR = 15 ms/750 ms/5 s, NA = 32, spectral bandwidth = 6000 Hz, number of points = 2048). For both experiments, TR was selected to maximize SNR of PCr signals^2^ according to the relaxation times reported at 7 T [14]. The total in vivo data acquisition time was 40 min.

All experiments were performed at a 7 T/68 cm MR scanner (Siemens Medical Solutions, Erlangen, Germany). The scanner is equipped with a gradient coil capable of reaching 80 mT/m in total, and 50 mT/m along each axis simultaneously. First- and second-order shims were optimized using the 3D gradient-echo shim.

### Data analysis

The first spectrum of each experiment was automatically phased, and the phased spectrum was used as the reference to phase the rest of averages by maximizing the correlation coefficients of the two spectra. Frequency drift correction was implemented using PCr as the reference peak. Individual spectra with SNR lower than 90% of the maximum SNR of the spectra with the respective *b*-value were discarded. SNR was calculated as the maximum signal intensity of PCr peak divided by the standard deviation of the noise without filtering. The spectra were then averaged and analyzed by LCModel for quantification. A basis set that contains 13 simulated spectra of ^31^P metabolites, including PCr, Pi, α-ATP, β-ATP, γ-ATP, phosphocholine (PC), phosphoethanolamine (PE), GPC, glycerophosphoethanolamine (GPE), membrane phospholipids (MP), nicotinamide adenine dinucleotide hydrogen (NADH), nicotinamide adenine dinucleotide oxidized (NAD +), and Pi (extra-cellular), was used [15]. The spectral quality was evaluated with SNR metric and PCr linewidth (FWHM). Metabolites that were quantified with Cramer–Rao bound (CRLB) lower than 20% at all *b*-values were selected for further analysis. ADCs were calculated by fitting the logarithmic geometric mean of metabolite concentrations measured at different polarities using a linear function. The goodness of the ADC fit was assessed with Pearson correlation coefficient R^2^. Fittings with R^2^ index lower than 0.50 were discarded due to poor fitting quality. The ADC values were reported for metabolites with more than half of the subjects being successfully measured and fit. Data analysis was implemented with Matlab R2021a and Python 3.8.8.

## Results

### In vitro results

To validate the sequence, we first performed the measurement of ADC of water in a phantom. The measured ADC of water was 1.935 × 10^–3^ mm^2^/s (*R*^2^ = 0.97). Then, we further validated the sequence by measuring ADCs of ^31^P metabolites with a phosphorous phantom. The measured ADCs of PCr, Pi, γ-ATP and α-ATP were (0.511, 0.598, 0.379, 0.368) × 10^–3^ mm^2^/s, respectively (*R*^2^ = 0.90, 0.99, 0.97, 0.98), as shown in Table 1.

**Table 1:** ADCs of water and ^31^P metabolites in vitro (10^–3^ mm^2^/s) measured at room temperature (~ 18 ℃)

Metabolites	Mean ADC	De Graaf [5]
Water	1.935	1.93 ± 0.02
PCr	0.511	0.48 ± 0.05
Pi	0.598	0.59 ± 0.09
γ-ATP	0.379	0.35 ± 0.05
α-ATP	0.368	

### In vivo results

**Fig. 2:**
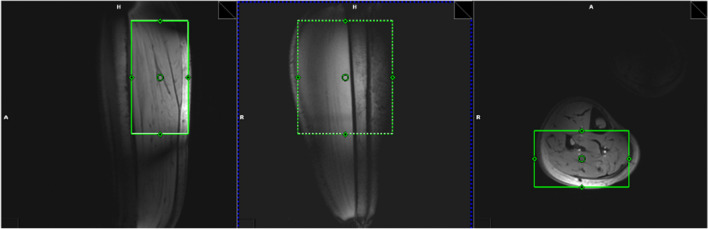
In vivo experiment voxel placement (in green) in the human calf muscle

**Fig. 3:**
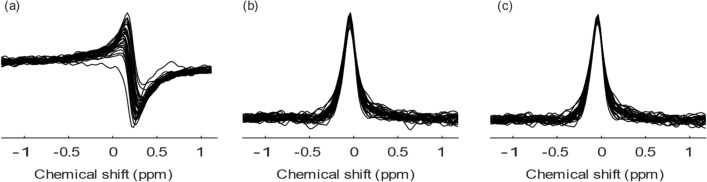
Spectra post-processing steps. **a** The measured PCr spectra; **b** the spectra after phase and frequency correction; **c** the spectra after removing scans with SNR lower than 90% of the maximum SNR of the spectra with the respective *b*-value, subject and direction

**Fig. 4:**
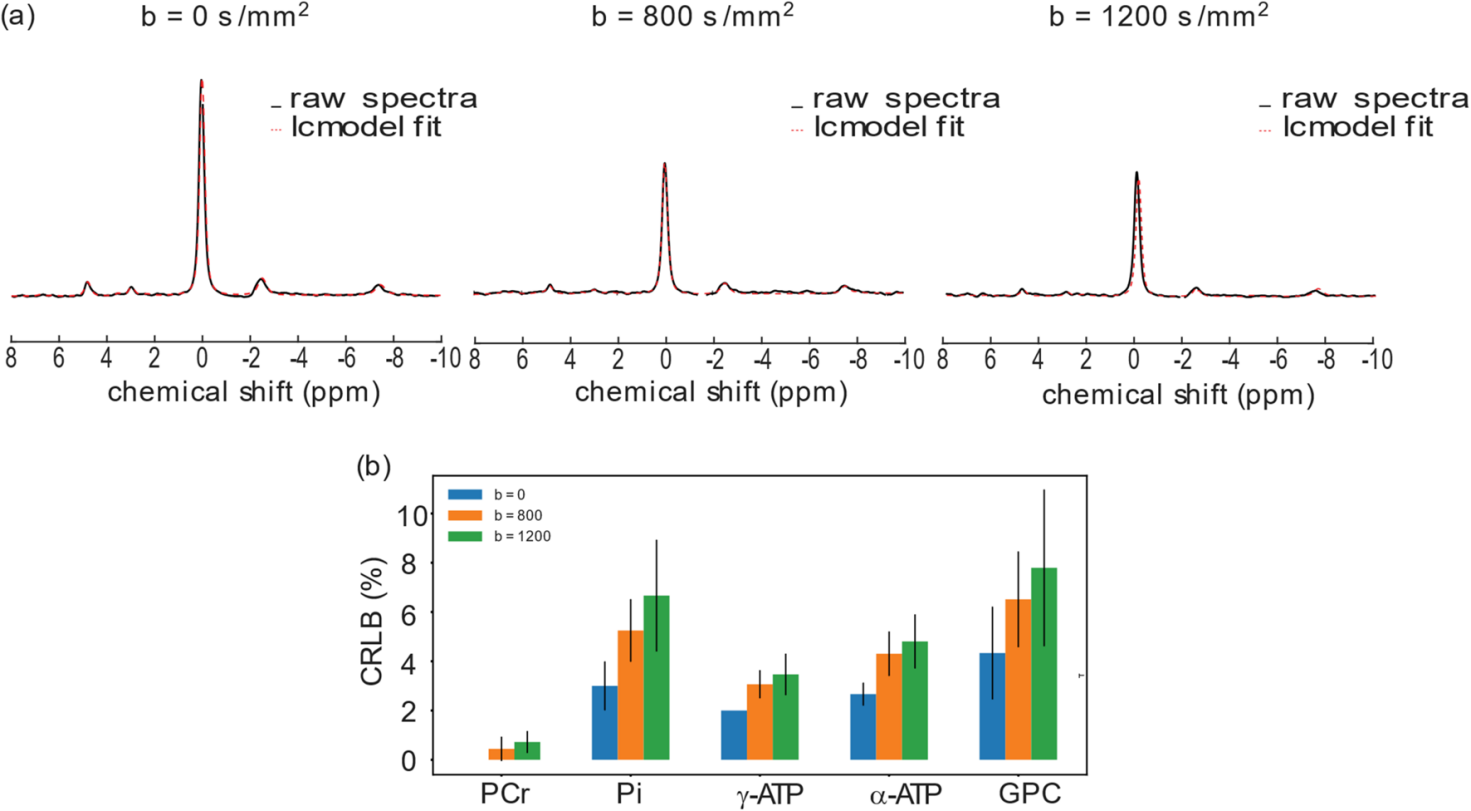
**a** Representative diffusion-weighted MR spectra acquired in the calf muscle of a volunteer, at all *b*-values along direction [1, 1, −0.5] and the corresponding spectral fits. **b** CRLBs of metabolites (mean and standard deviation) at different *b*-values

The in vivo ADC values of Pi, PCr, ATP, and GPC were measured in the human calf muscle. The voxel was placed as shown in Fig. 2. The post-processing steps including phase and frequency alignment, and low SNR spectra removal are illustrated in Fig. 3. High spectral SNR was achieved at all *b*-values (108 ± 28, 72 ± 23, 62 ± 23 for b = 0, 800, 1200 s/mm^2^, respectively), with average linewidth of PCr peak being around 13.7 Hz. PCr was reliably quantified at all *b*-values with mean CRLB less than 1%, while γ-ATP, α-ATP, Pi and GPC with mean CRLB less than 10% (Fig. 4b). Figure 4a shows representative localized DW spectra along directions of [−0.5, 1, 1]. As the *b*-value increased, the signal intensity decay of PCr, γ-ATP and α-ATP can be visually observed. The signal intensity of all four visible peaks decreased monotonically with the *b*-value. The individual linear fit plots and *R*^2^ are shown in Fig. S1 and Table S1–S4. The mean ADCs of PCr, Pi, γ-ATP and GPC were (0.24 ± 0.02, 0.43 ± 0.14, 0.15 ± 0.04, 0.40 ± 0.09) × 10^–3^ mm^2^/s, respectively with average R^2^ being 0.97, 0.92, 0.81, 0.88 (Table 2). With the one-way ANOVA analysis as well as the Tukey–Kramer post-hoc analysis, significant difference of ADCs in different directions was found for PCr (0.2950 mm^2^/s for [1, −0.5, 1], 0.232 mm^2^/s for [−0.5, 1, 1], and 0.194 mm^2^/s for [1, 1, −0.5], *p* = 0.002), mainly driven by the differences between direction [1, −0.5, 1] and [−0.5, 1, 1] (*p* = 0.040) and that between [1, −0.5, 1] and [1, 1, −0.5] (*p* = 0.002).

**Table 2:** ADCs of ^31^P metabolites in vivo and reference values with similar diffusion time (10^–3^ mm^2^/s)

Metabolites	Nb. subjects	Mean ADC	R^2^	Gabr et al. [4] (human)	De Graaf [5] (rat)	Moonen et al. [7] (rat)
Pi	4	0.43 ± 0.14	0.92 ± 0.05	–	–	–
PCr	6	0.24 ± 0.02	0.97 ± 0.02	0.40 ± 0.06	~ 0.24	0.33
ATP	4	0.15 ± 0.04	0.81 ± 0.04	–	~ 0.18	–
GPC	4	0.40 ± 0.09	0.88 ± 0.09	–		–

## Discussion

In this study, a DW-STEAM sequence with short TE was implemented to measure the ADC of ^31^P metabolites in the human calf muscle at 7 T. To the best of our knowledge, this is the first study reporting the ADCs of ATP, Pi, and GPC, and the second study reporting the ADC of PCr in human.

The ADCs of PCr and ATP in human calf muscle measured in the current study (~ 0.24 × 10^–3^ mm^2^/s and 0.15 × 10^–3^ mm^2^/s, respectively) were in a good agreement with the values reported in rat skeletal muscles [5, 7]. However, the ADC of PCr was lower than that reported by Gabr et al. [4] in human calf muscles under similar diffusion time (~ 755 ms). The disagreement could lie in the differences during the post-processing process. Diffusion MRS is very sensitive to motions occurred during the application of diffusion gradients, which could lead to phase dispersion and additional signal loss and thus the overestimation of ADC. When the SNR is high enough, scan-to-scan phase correction could improve the ADC measurement accuracy [16, 17]. Due to the low SNR of the individual scan in the study performed by Gabr et al. [4] at 3T, no phase correction was implemented and thus the PCr’s ADC was likely to be overestimated [4]. In the current study, scan-to-scan phase and frequency correction were implemented prior to spectra averaging to mitigate the problem. In addition, eddy current effects could also lead to additional spectra dephasing. In our work, eddy currents were not detected in phantom experiments. The measured ADC of Pi was (0.43 ± 0.14) × 10^–3^ mm^2^/s, higher than that reported by K. Yoshizaki et al. in bullfrog muscles ex vivo at room temperature (24 ℃) [10]. In their study, spin echo sequence was implemented with diffusion time being ~ 20 ms, with ADC being ~ 0.33 × 10^–3^ mm^2^/s. This discrepancy could be due to the different experimental conditions (human vs bullfrogs and in vivo vs ex vivo, respectively), including also the difference in temperature (37 ℃ in vivo vs 24 ℃ ex vivo).

Since muscle fibers are highly directional, ADCs of metabolites may be anisotropic. Indeed, the ADC of PCr along the direction [1, −0.5, 1] was significantly larger than the other two directions, especially when compared with that along the direction [1, 1, −0.5]. This is coherent with the physiological conditions. Since the fiber direction roughly aligned with the direction [0, 0, 1], the directions with more contributions from the fiber elongation direction was supposed to have larger diffusion coefficients. Anisotropy was not detected for other metabolites, which might be due to relatively low SNR.

Noteworthily, considering the bandwidth of the RF pulses used and the chemical shift of the metabolites, the chemical shift displacement errors in one direction for Pi, γ-ATP and GPC are 11%, 9%, and 18%, respectively, indicating a 71%, 75% and 56% voxel overlap with that of PCr. In the case of large measurement voxel, removing the localization gradients and using the direct coil localization could further improve the SNR by allowing the use of a shorter echo time, and eliminate the chemical shift displacement error.

Measuring the ADC of ATP in vivo is challenging due to the low concentration, short T_2_ relaxation time, and J-coupling effects. It is even more difficult in humans due to the restriction on maximum achievable *b*-values posed by the allowed gradient amplitudes and TE duration. To achieve short enough echo time to measure the ADC of ATP, STEAM sequence was implemented rather than spin echo sequence. To reach a high *b*-value for ADC measurements, a long TM and a long diffusion time has to be used due to the limited diffusion gradient amplitude and duration during the TE. Therefore, the current protocol prevents the measurement of ADC of ATP at short diffusion time (< 100 ms), which limits the possibility to study the cellular environmental restriction of ATP. An approach to refocus J-evolution of ATP was proposed in a study with a frequency-selective DW-STEAM on a preclinical MR scanner [5]. Briefly, frequency-selective SSAP pulses were applied as the 2nd and 3rd excitation pulse to selectively excite α- and γ-ATP, while having no influence on β-ATP, J-evolution of α- and γ-ATP resonances could then be refocused [5]. SSAP is a type of BIR-4 pulse and to reach sufficient excitation bandwidth, a large peak B_1_ is commonly required, which limits its implementation on a clinical scanner. Furthermore, due to the frequency-selective nature of the pulse, the localization gradient could not be applied simultaneously. A similar J-refocused approach was implemented to measure the ADC of lactate [18] in the mouse brain. The spectrally selective pulse was applied in a DW-SE-LASER sequence where the diffusion block and localization block were separated. Taken into account the short T_2_ of ATP, future studies could implement J-refocused DW module with coil localization for studying the cellular environment and diffusion anisotropy of ATP in vivo. To study the ADC of PCr in specific muscle compartment, separate localization module such as image-selected in vivo spectroscopy (ISIS) localization [19, 20] could be combined with the DW module.

The PCr/creatine kinase energy shuttle is the primary hypothesis for cellular energy transport. In this hypothesis, energy is transferred from ATP to PCr, and PCr carries the high-energy phosphates to the energy utilization sites by diffusion and forms ATP there [21]. By measuring the ADC of PCr in the human calf muscle with different diffusion times and comparing it with creatine kinase reaction rates, Gabr et al. explored the cellular environment of PCr in the human muscle and proposed that the energy shuttle hypothesis might not be obligatory for energy transport between mitochondria and myofibrils [4]. In our study, it was measured that PCr diffuses in cells with an ADC of 0.24 × 10^–3^ mm^2^/s, which means that within the half-life (2.6 s) of PCr in the CK reaction (reaction rate: 0.27 s^−1^) [4, 22], PCr could diffuse an average distance of ~ 61 μm. This distance is comparable with that derived by Gabr et al., and is much longer than a mitochondria-myofibril distance of up to ~ 2 μm [4, 23]. This result further validated the argument that PCr diffusion is not a limiting factor for the supply of PCr to CK reactions [4, 10] in the human muscle. In the current study, a large voxel size was implemented in order to measure the ADC of ATP. Based on an SNR analysis, current results suggested that the SNR for PCr was sufficient to potentially allow PCr ADC measurement in smaller voxels for future investigations in specific muscle compartments.

We conclude that it is feasible to measure the ADC of four major ^31^P metabolites (PCr, ATP, Pi and GPC) in vivo in the human calf muscle at 7 T. This study paves the way to investigate ^31^P metabolite diffusion properties in health and disease on the clinical MR scanner.

## Supplementary Information

Below is the link to the electronic supplementary material.

Supplementary file1 (PDF 433 KB)

## Data Availability

The data of this study are available from the corresponding author upon reasonable request.

## References

[1] Rietzler A, Steiger R, Mangesius S (2022). Energy metabolism measured by 31P magnetic resonance spectroscopy in the healthy human brain. J Neuroradiol.

[2] van der Kemp WJM, Stehouwer BL, Runge JH (2016). Glycerophosphocholine and glycerophosphoethanolamine are not the main sources of the *In vivo *31P MRS phosphodiester signals from healthy fibroglandular breast tissue at 7T. Front Oncol.

[3] Klein J (2000). Membrane breakdown in acute and chronic neurodegeneration: focus on choline-containing phospholipids. J Neural Transm.

[4] Gabr RE, El-Sharkawy AMM, Schär M, Weiss RG, Bottomley PA (2011). High-energy phosphate transfer in human muscle: diffusion of phosphocreatine. Am J Physiol Cell Physiol.

[5] de Graaf RA, van Kranenburg A, Nicolay K (2000). *In vivo* 31P-NMR diffusion spectroscopy of ATP and phosphocreatine in rat skeletal muscle. Biophys J.

[6] Hubley MJ, Moerland TS, Rosanske RC (1995). Diffusion coefficients of atp and creatine phosphate in isolated muscle: pulsed gradient 31p nmr of small biological samples. NMR Biomed.

[7] Moonen CT, van Zijl PC, Le Bihan D, DesPres D (1990). *In vivo* NMR diffusion spectroscopy: 31P application to phosphorus metabolites in muscle. Magn Reson Med.

[8] Kinsey ST, Locke BR, Penke B, Moerland TS (1999). Diffusional anisotropy is induced by subcellular barriers in skeletal muscle. NMR Biomed.

[9] van Gelderen P, DesPres D, van Zijl PC, Moonen CT (1994). Evaluation of restricted diffusion in cylinders. Phosphocreatine in rabbit leg muscle. J Magn Reson B.

[10] Yoshizaki K, Watari H, Radda GK (1990). Role of phosphocreatine in energy transport in skeletal muscle of bullfrog studied by 31P-NMR. Biochim Biophys Acta BBA - Mol Cell Res.

[11] Bogner W, Chmelik M, Schmid AI, Moser E, Trattnig S, Gruber S (2009). Assessment of 31P relaxation times in the human calf muscle: a comparison between 3T and 7T *in vivo*. Magn Reson Med.

[12] Le Roux P. *Introduction to the Shinnar-Le Roux Algorithm*; 1995. doi: 10.13140/RG.2.2.34523.49448

[13] Pauly J, Le Roux P, Nishimura D, Macovski A (1991). Parameter relations for the Shinnar-Le Roux selective excitation pulse design algorithm (NMR imaging). IEEE Trans Med Imaging.

[14] Valette J, Giraudeau C, Marchadour C (2012). A new sequence for single-shot diffusion-weighted NMR spectroscopy by the trace of the diffusion tensor. Magn Reson Med.

[15] Cuenoud B, Ipek Ö, Shevlyakova M (2020). Brain NAD is associated with ATP energy production and membrane phospholipid turnover in humans. Front Aging Neurosci.

[16] Ellegood J, Hanstock CC, Beaulieu C (2005). Trace apparent diffusion coefficients of metabolites in human brain using diffusion weighted magnetic resonance spectroscopy. Magn Reson Med.

[17] Deelchand DK, Auerbach EJ, Marjańska M (2018). Apparent diffusion coefficients of the five major metabolites measured in the human brain *in vivo* at 3T. Magn Reson Med.

[18] Mougel E, Malaquin S, Valette J (2022). Assessing potential correlation between T2 relaxation and diffusion of lactate in the mouse brain. Magn Reson Med.

[19] Ordidge RJ, Connelly A, Lohman JAB (1986). Image-selected in vivo spectroscopy (ISIS). A new technique for spatially selective NMR spectroscopy. J Magn Reson.

[20] Valkovič L, Bogner W, Gajdošík M (2014). One-dimensional image-selected *in vivo* spectroscopy localized phosphorus saturation transfer at 7T. Magn Reson Med.

[21] Bessman SP, Geiger PJ (1981). Transport of energy in muscle: the phosphorylcreatine shuttle. Science.

[22] Bottomley PA, Ouwerkerk R, Lee RF, Weiss RG (2002). Four-angle saturation transfer (FAST) method for measuring creatine kinase reaction rates *in vivo*. Magn Reson Med.

[23] Gosker HR, Hesselink MKC, Duimel H, Ward KA, Schols AMWJ (2007). Reduced mitochondrial density in the vastus lateralis muscle of patients with COPD. Eur Respir J.

