# Supplementary material for: Apparent diffusion coefficients of 31P metabolites in the human calf muscle at 7 T
Source: MAGMA. 2023 Feb 8;36(2):309–15. doi: 10.1007/s10334-023-01065-3 (PMC10140108; doi:10.1007/s10334-023-01065-3)
Supplement: Supplementary file 1 — Supplementary file1 (PDF 433 KB) [file 10334_2023_1065_MOESM1_ESM.pdf]

Magnetic Resonance Materials in Physics, Biology and Medicine

Apparent diffusion coefficients of 31P metabolites in the human calf muscle at 7T

Supplementary Material

Zhiwei Huang[1,2], Giulio Gambarota[3], Ying Xiao[1,2], Daniel Wenz[1,2] and Lijing Xin[1,2]

1. Animal imaging and technology core (AIT), Center for Biomedical Imaging (CIBM), Ecole Polytechnique Fédérale de Lausanne, Lausanne, Switzerland
2. CIBM Center for Biomedical Imaging, Switzerland
3. Faculty of Pharmacy, University of Rennes, Rennes, France

Corresponding author:

Lijing Xin, Ph.D., EPFL CIBM-AIT, Station 6, CH-1015 Lausanne, Switzerland

lijing.xin@epfl.ch

Tel.: + 41 21 693 0597

Fax: + 41 21 693 7960

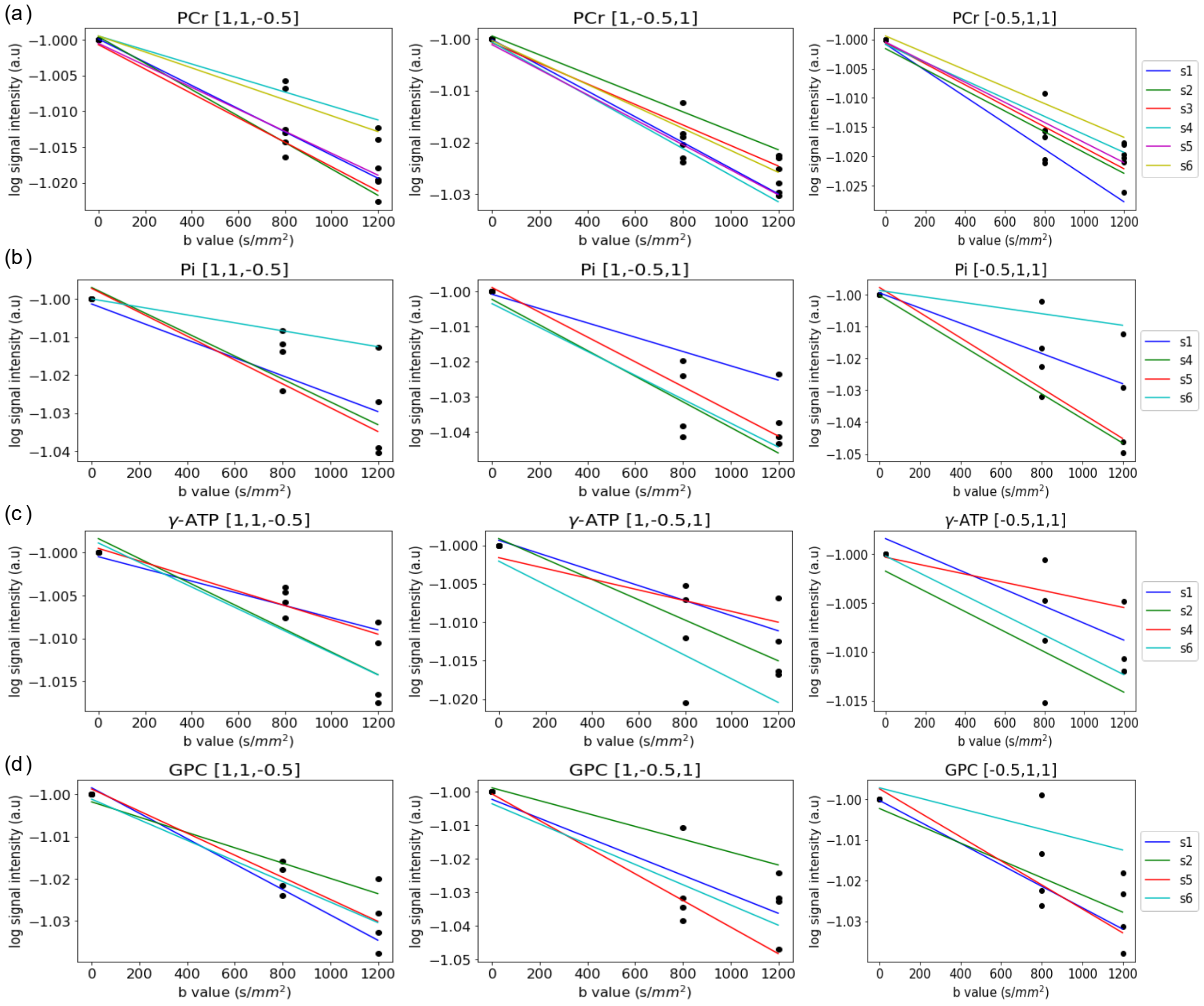

Figure S1. ADC linear fitting plots of phosphorous metabolites for individual subjects along directions [1, 1, -0.5], [1, -0.5, 1], [-0.5, 1, 1]. For the simplicity of visualization, each individual was normalized by dividing the log signal intensity by that of b = 0 s/mm^2.

Table S1. R^2 of the ADC fitting of PCr

R^2 (PCr)	Subject 1	Subject 2	Subject 3	Subject 4	Subject 5	Subject 6
[1, -0.5, 1]	0.999	0.982	0.973	0.989	0.962	0.996
[-0.5, 1, 1]	0.977	0.872	0.981	0.946	0.988	0.970
[1, 1, -0.5]	0.992	0.990	0.972	0.947	0.980	0.959

Table S2. R^2 of the ADC fitting of Pi

R^2 (Pi)	Subject 1	Subject 4	Subject 5	Subject 6
[1, -0.5, 1]	0.967	0.930	0.984	0.832
[-0.5, 1, 1]	0.990	0.999	0.943	0.718
[1, 1, -0.5]	0.947	0.840	0.870	1.000

Table S3. R^2 of the ADC fitting of γ-ATP

R^2 (γ-ATP)	Subject 1	Subject 2	Subject 4	Subject 6
[1, -0.5, 1]	0.922	0.924	0.505	0.747
[-0.5, 1, 1]	0.612	0.653	0.907	0.994
[1, 1, -0.5]	0.924	0.776	0.935	0.872

Table S4. R^2 of the ADC fitting of GPC

R^2 (GPC)	Subject 1	Subject 2	Subject 5	Subject 6
[1, -0.5, 1]	0.892	0.936	0.995	0.791
[-0.5, 1, 1]	0.997	0.819	0.878	0.527
[1, 1, -0.5]	0.951	0.851	0.956	0.962

Table S5. Checklist for a single voxel 31P-MRS study

1.	Hardware	
a.	Field strength [T]	7T
b.	Manufacturer	Siemens Medical Solutions, Erlangen, Germany
c.	Model	Magnetom 7T (VB17)
d.	RF coils: nuclei (transit/receive), number of channels, type, body part	Home-built single loop 31P (diameter = 7cm), two-loop 1H (diameter = 10cm) surface coil
e.	Additional hardware	N/A

2.	Acquisition	
a.	Pulse sequence	STEAM
b.	Volume of interest and VOI locations	Single voxel placed in the human calf muscle
c.	Nominal VOI size	100 mm x 120 mm x 60 mm
d.	Repetition time (TR), echo time (TE)	TR = 5 s, TE = 15 ms
e.	Total number of excitations or acquisitions per spectrum (NA)	In total 3 excitations per number of acquisition, and NA=32 per b value per direction
f.	Additional sequence parameters (spectral width in Hz, number of spectral points, frequency offsets)	The mixing time TM = 750 ms, b values = 0, 800, 1200 s/mm^2, directions are ± [1, 1, -0.5], ± [1, -0.5, 1], ± [-0.5, 1, 1], spectral bandwidth is 6 kHz, 2048 complex points
g.	Water suppression method	N/A
h.	Shimming method, reference peak, and threshold for "acceptance of shim" chosen	1st and 2nd order shims with 3D gradient-echo shim. The reference peak is PCr peak.
i.	Triggering or motion correction method (respiratory, peripheral, cardiac triggering, incl. device used and delays)	Subjects were instructed to put their leg on the surface coil, and two pads were put near their legs to help immobilize them.

3.	Data analysis methods and outputs	
a.	Analysis software	31P MR spectrum were post-processed with custom-built Matlab R2021b scripts. The processed spectra were quantified with LCModel. Apparent diffusion coefficients (ADC) were fitted with Python 3.8.8, using *scipy.optimize.curve_fitting* function.
b.	Processing steps deviating from quoted reference or product analysis software (vendor, version)	Frequency and phase correction were implemented in the time domain, and low-SNR spectra removal were implemented based on the maximum PCr signal intensity.
c.	Output measure (e.g, absolute concentration, institutional units, ratio), processing steps deviating from quoted reference or product	Absolute concentrations and ADC values
d.	Quantification references and assumptions, fitting model assumptions	No reference was used. The LCModel output of absolute concentration with arbitrary unit was used for ADC fitting. ADC values were fitted based on the assumption of mono-exponential decay.

4.	Data quality	
a.	Reported variables (SNR, linewidth (with reference peaks))	SNR was calculated by dividing the maximum PCr intensity by the standard deviation of noise signals within 38 to 50 ppm. Linewidth was calculated as the FWHM of the PCr peak.
b.	Data exclusion criteria	Individual spectra with SNR lower than 90% of the maximum SNR of the spectra with the same b value and direction were discarded. Metabolites with CRLB higher than 20% at any b value were discarded. ADC fitting with R^2 lower than 0.50 were discarded.
c.	Quality measures of postprocessing model fitting (e.g, CRLB, goodness of fit, AD of residual)	CRLB was used for quantification quality measures, R^2 was used for ADC fitting quality measures.
d.	Sample spectrum	Figure 4
